# PARPACT – Paramedic Palliative Care Test: Validierung eines Tests für palliatives Wissen und Selbstwirksamkeitserwartung bei Mitarbeiter*innen im Rettungswesen

**DOI:** 10.1007/s00482-021-00587-w

**Published:** 2021-09-29

**Authors:** D. Chwallek, A. Schweda, M. Neukirchen, J. Hense, J. Schwartz, B. Mallmann, M. Teufel, M. Schuler, Mitra Tewes

**Affiliations:** 1 Westdeutsches Tumorzentrum, Innere Klinik (Tumorforschung), Universitätsklinikum Essen, 45122 Essen, Deutschland; 2 Klinik für Psychosomatische Medizin und Psychotherapie, LVR-Klinikum Essen, Universität Duisburg-Essen, 45147 Essen, Deutschland; 3 Klinik für Anästhesiologie, Interdisziplinäres Zentrum für Palliativmedizin, CIO Düsseldorf, Universitätsklinikum Düsseldorf, Heinrich Heine Universität, 40225 Düsseldorf, Deutschland; 4 Universitätsklinikum Essen, 45122 Essen, Deutschland; 5 Partnerstandort Universitätsklinikum Essen, Deutsches Konsortium für Translationale Krebsforschung (DKTK), Essen, Deutschland

**Keywords:** Palliativversorgung, Rettungsdienst, Messinstrument, Palliativbildung, Validierung, Palliative Care, Emergency services, measurement tool, Palliative education, Validation

## Abstract

**Hintergrund:**

Angesichts der Vielzahl von Palliativpatienten, die vom Rettungsdienst versorgt werden, spielen Aus- und Weiterbildung in palliativmedizinischen Themen eine immer größere Rolle. Zur Verbesserung der Entscheidungsfindung in Rettungssituationen wurde in vielen Städten ein Palliativ- bzw. Notfallausweis eingeführt.

**Ziel der Arbeit:**

Um den Erfolg von Bildungsmaßnahmen und den Effekt des Palliativ- bzw. Notfallausweises zu überprüfen, wurde ein Fragebogen zur Ermittlung von palliativem Wissen und palliativer Selbstwirksamkeitserwartung im Rettungsdienst entwickelt und validiert.

**Material und Methoden:**

Die Entwicklung und Inhaltsvalidierung erfolgte mithilfe eines Delphi-Prozesses. Zur Konstruktvalidierung wurde die Faktorenanalyse genutzt. Die Kriteriumsvalidität wurde anhand von 22 speziell in Palliative Care geschulten Pflegekräften überprüft. Die Reliabilität wurde mittels Cronbachs α als Maß der internen Konsistenz ermittelt.

**Ergebnisse:**

291 von 750 Rettungsdienstmitarbeitern nahmen an der freiwilligen Befragung teil. Nach Abschluss des Delphi-Prozesses bestand Konsens, dass die wichtigen Themen Schmerz, Dyspnoe, Sedierung, Sterbebegleitung, Sterbehilfe und rechtliche Aspekte im Fragebogen abgedeckt sind. Das Ergebnis der Faktorenanalyse sprach für eine 6‑Faktoren-Lösung. Bei der Kriteriumsvalidierung zeigte sich ein signifikanter Unterschied im palliativen Wissen zwischen den Palliative-Care-Pflegekräften (M_Rang_ 289,73) und den Rettungsdienstmitarbeitern (M_Rang_ 146,97, U = 281.000, r = 0,40, *p* < 0,001). Cronbachs α lag für die Wissensfragen bei 0,70 und für die Subskala der palliativen Selbstwirksamkeitserwartung bei 0,82.

**Diskussion:**

Mit dem Paramedic Palliative Care Test (PARPACT) liegt ein validiertes Messinstrument zur Überprüfung von Bildungsmaßnahmen im Rettungsdienst vor.

## Abstract (en)

**Background:**

Given the large number of palliative patients cared for by the emergency services, education and training in palliative care topics are playing an increasingly important role. To support decision-making in an emergency setting a palliative or emergency card has been introduced in many cities.

**Objectives:**

To assess the success of educational interventions and the effect of the palliative or emergency card, a questionnaire was developed and validated to determine palliative knowledge and palliative self-efficacy expectations in the emergency services.

**Materials and methods:**

A Delphi process was applied for development and content validation. Factor analysis was used for construct validation. Criterion validity was assessed with the help of 22 nurses specially trained in palliative care. Reliability was determined using Cronbach’s alpha as a measure of internal consistency.

**Results:**

In all, 291 of 750 paramedics participated in the voluntary survey. After completion of the Delphi process, there was consensus that the important topics of pain, dyspnea, sedation, end-of-life care, euthanasia, and legal aspects were covered in the questionnaire. Factor analysis was in favor of a six-factor solution. Criterion validation revealed a significant difference in palliative knowledge between palliative care nurses (M_Rang_ 289.73) and paramedics (M_Rang_ 146.97, U = 281.000, r = 0.40, *p* < 0.001). Cronbach’s alpha was 0.70 for the knowledge questions and 0.82 for the palliative care self-efficacy expectancy subscale.

**Conclusions:**

The Paramedic Palliative Care Test (PARPACT) is a validated measurement tool for testing educational interventions in paramedicine.

3–10 % aller Notfalleinsätze im Rettungswesen sind Einsätze bei Palliativpatienten [22]. Eine frühere Studie konnte zeigen, dass sich viele Rettungsdienstmitarbeiter in Einsätzen bei Palliativpatienten und im Umgang mit Patientenverfügungen unsicher fühlen [27]. Zwischenzeitlich haben einige Rettungsdienstmitarbeiter durch die Notfallsanitäterausbildung mehr Kompetenzen im Bereich der Palliativmedizin erlangt und Unterstützung durch die Einführung von Palliativ- bzw. Notfallausweisen erhalten. Mithilfe eines validierten Fragebogens können der Erfolg neuer Bildungsmaßnahmen oder Effekte des Palliativ- bzw. Notfallausweises ermittelt werden.

## Hintergrund

Auf den ersten Blick scheint es kaum eine Verbindung zwischen der Notfallmedizin mit dem Ziel der Lebenserhaltung und der Palliativmedizin mit dem Ziel der symptomatischen Linderung und Verbesserung der Lebensqualität ohne Lebensverlängerung um jeden Preis zu geben. Dank der spezialisierten ambulanten palliativen Versorgung (SAPV) können mittlerweile viele Palliativpatienten zu Hause versorgt werden [10]. Kommt es zu Symptomexazerbationen oder sind Angehörige mit Notfallsituationen überfordert, wird aber häufig der Rettungsdienst verständigt, der sich nun in kurzer Zeit einen Überblick verschaffen und über die weitere Versorgung des Patienten entscheiden muss [21, 28]. Dies verlangt ein hohes Maß an Kompetenz und Erfahrung. In der bisherigen Rettungsassistentenausbildung nach Rettungsassistentengesetz (RettAssG; [14]) spielten palliativmedizinische Ausbildungsinhalte eher eine untergeordnete Rolle, dabei wurde bereits schon früher auf die Bedeutung von palliativmedizinischen Schulungen für eine kompetente Versorgung von Palliativpatienten hingewiesen [22, 25, 27]. In der der Rettungsassistentenausbildung folgenden Notfallsanitäterausbildung werden nach dem Notfallsanitätergesetz (NotSanG; [19]) psychosoziale und heilkundlerische Kompetenzen gar explizit gefordert [12]. Konzepte für palliativmedizinische Fortbildungsinhalte liegen bereits vor. Die Deutsche Gesellschaft für Palliativmedizin (DGP) hat eigens in einer Arbeitsgruppe eine „Kompetenzbasierte berufsgruppenunabhängige Matrix zur Erstellung von Curricula für die Weiterbildung curricularer Bildungsinhalte in Palliative Care/Palliativmedizin“ (KoMPaC) entwickelt [30]. Auch mit der Einführung von Palliativausweisen/Notfallausweisen in vielen deutschen Städten und Landkreisen soll der Rettungsdienst besonders bei rechtlichen Fragen und im Umgang mit Patientenverfügungen unterstützt werden [13, 26]. Um den Erfolg von Bildungsmaßnahmen oder den Effekt des Palliativ- bzw. Notfallausweises zu evaluieren, sind geeignete Instrumente zur Messung von palliativem Wissen und palliativer Selbstwirksamkeitserwartung notwendig. Aus diesem Grund erfolgte mithilfe einer Expertengruppe die Entwicklung und anschließende Validierung des Paramedic Palliative Care Test (PARPACT). Als Vorbild bei der Entwicklung des Paramedic Palliative Care Test (PARPACT) dienten der Bonner Palliativwissenstest (BPW; [18]) und der Palliativkompetenztest für Ärzte (PKT; [16]). Beide Tests beinhalten Items zur palliativen Wissensabfrage sowie zur spezifisch palliativen Selbstwirksamkeitserwartung und wurden vorwiegend zur Evaluation von Bildungsmaßnahmen entwickelt. Da allerdings mehrere Items sowohl des BPW als auch des PKT für Ärzte für einen Test im Rettungsdienst ungeeignet sind, wurde ein neues Messinstrument entwickelt. Die Ergebnisse dieser Entwicklung und Validierung präsentieren wir in der vorliegenden Arbeit.

## Material und Methodik

### Delphi-Prozess und Befragung

Die Expertenrunde setzte sich aus vier Palliativmedizinern, zwei Notfallmedizinern, drei Rettungsdienstmitarbeitern, zwei SAPV-Mitgliedern und einem Psychologen zusammen. Bei Auswahl der Experten wurde Wert auf Vorerfahrungen gelegt. So besaß einer der Palliativmediziner ebenfalls mehrjährige Erfahrungen als Notfallmediziner. Weiterhin war einer der Notfallsanitäter, einer der Rettungsdienstmitarbeiter wie auch ein SAPV-Mitglied an der Entwicklung des Palliativausweises/Notfallausweises beteiligt. Darüber hinaus war ein weiterer Palliativmediziner lange Zeit in der SAPV und als Chirurg tätig. In vier Runden wurden die Bestandteile und Inhalte des Testinstruments definiert. Die Experten waren sich einig, dass Items zu den Themen Schmerz, Sedierung, Dyspnoe, Sterbebegleitung, Sterbehilfe und rechtliche Aspekte unverzichtbare Bestandteile eines Instruments zur Messung von palliativem Wissen sein müssten. Als Skala wurde für alle Items eine 4‑Punkte-Likert-Skala mit den Kategorien (stimmt nicht, stimmt kaum, stimmt eher, stimmt) verwendet.

Um die Praxistauglichkeit des Messinstruments für die Rettungsdienstmitarbeiter noch zu verbessern, wurde außerdem ein Fallbeispiel entwickelt, mit dem ebenfalls palliatives Wissen gemessen werden sollte.

Auch Fragen zum Schulungsbedarf bezüglich verschiedener palliativmedizinischer Themen wurden dem Fragebogen hinzugefügt.

Nach der Pilotierung wurde der Fragebogen in der zweiten Stichprobe Rettungsdienstmitarbeitern zweier ländlich und zweier städtisch strukturierter Rettungsdienstbereiche vorgelegt. Die freiwillige Befragung der Rettungsdienstmitarbeiter erfolgte im Rahmen von regelmäßig stattfindenden Schulungen in den Rettungsdienstbereichen.

Insbesondere zur Messung der Kriteriumsvalidität wurde der Fragebogen zusätzlich von 22 Palliative-Care-Pflegekräften auf freiwilliger Basis beantwortet. Die Daten der Pflegekräfte wurden auch bei der Berechnung der Reliabilität, Trennschärfe, Faktorenanalyse und konvergenten Validität aus Gründen der Stichprobenmaximierung berücksichtigt.

### Statistische Kennwerte und Validierungsprozess

Die Auswertung der Fragebögen und die statistischen Berechnungen erfolgten unter Verwendung von SPSS Statistics Version 25 (IBM, Armonk, NY, USA) und R 3.6.3 (https://www.r-project.org). Um eine gute Auswertungsobjektivität zu erreichen, wurden für die Befragung geschlossene Fragen verwendet. Außerdem wurden Items mit fehlenden Werten ausgeschlossen. Vor Auswahl der für die Validierung geeigneten Tests wurden die Items mittels Kolmogorov-Smirnov-Test auf Normalverteilung überprüft. Zur Überprüfung der Konstruktvalidität des Messinstrumentes wurde eine explorative Faktorenanalyse durchgeführt. Hierfür wurden die Hauptachsenanalyse und die Oblimin-Rotation mit einem δ‑Wert von 0 gewählt. Als Extraktionskriterium für die Faktoren wurde die von vielen Autoren [2, 5, 29] empfohlene Parallelanalyse berücksichtigt. Anschließend wurden die Mittelwerte der jeweiligen Items eines Faktors addiert und nach Spearman miteinander korreliert. Für die konvergente Validierung wurde eine Spearman-Korrelation aller Items zur Messung von palliativer Selbstwirksamkeitserwartung mit der bereits validierten allgemeinen SWE-Skala nach Schwarzer und Jerusalem [9] berechnet. Weiterhin wurden die Testergebnisse im palliativen Wissen von allen befragten Rettungsdienstmitarbeitern mit den Testergebnissen von Palliative-Care-Pflegekräften mittels Whitney-U-Test verglichen. Als Hypothese wurde die Erwartung formuliert, dass die Pflegekräfte im palliativen Wissen höhere Scores erreichen als die Rettungsdienstmitarbeiter. Durch diesen Vergleich wurde die Kriteriumsvalidität des Messinstruments bewertet. Als Maß der Effektstärke diente für die konvergente Validität und Kriteriumsvalidität die Interpretation von r nach Cohen [4]. Die Reliabilität wurde mittels interner Konsistenz durch Berechnung von Cronbachs α für die Subskalen palliatives Wissen und palliative Selbstwirksamkeitserwartung ermittelt. Ferner wurde die Trennschärfe als Ausdruck der Korrelation der einzelnen Items mit dem Gesamttestwert berechnet. Ein Testwert < 0,3 wurde hierbei als gering angesehen.

## Ergebnisse

Zur Pilotierung wurde der Fragebogen 16 Rettungsdienstmitarbeitern vorgelegt. Für 13 Rettungsdienstmitarbeiter waren die Items sehr verständlich formuliert und 14 Rettungsdienstmitarbeiter hielten die Fragen für ihre Tätigkeit im Rettungsdienst für sehr relevant.

Es nahmen insgesamt 291 von 750 Rettungsdienstmitarbeitern (249 Männer, 37 Frauen, 1 Divers, 4 keine Angaben) im Alter von 35 ± 9 Jahren (Mittelwert ± Standardabweichung) an der freiwilligen Befragung teil. Die Rekrutierungsquote betrug somit 39 %. 113 aller Befragten (38,8 %) hatten die Ausbildung zum Notfallsanitäter abgeschlossen. Die durchschnittliche Berufserfahrung im Rettungsdienst betrug 11 ± 8 Jahre bzw. im Median 10 Jahre (1–35 Jahre).

Von den Fragen zum palliativen Wissen wurden durchschnittlich 4,4 % nicht beantwortet oder die Antworten waren ungültig. Bei den Items zur palliativen Selbstwirksamkeitserwartung waren durchschnittlich 0,6 % der Antworten fehlend bzw. ungültig. Wie häufig die jeweiligen Items von den Rettungsdienstmitarbeitern nicht beantwortet wurden, ist in Tab. 1 aufgelistet.

**Table 1:** 

	Von den RDM nicht beantwortet, *n* (%)	Trennschärfe	Reliabilität „α if item deleted“
**Wissen und Entscheidung**			
1. Patienten mit lebensbedrohlichen Erkrankungen sollte die Wahrheit nicht vorenthalten werden, damit sie sich auf ihren Tod vorbereiten können (BPW)	3 (1,0)	0,06	0,70
2. Bei vorbestehender Therapie mit Opioiden ist die zusätzliche Gabe von Sedativa (z. B. Midazolam, Diazepam) wegen Gefahr der Atemdepression kontraindiziert^a^	29 (10)^b^	0,28	0,68
3. Familienmitgliedern sollte die Anwesenheit am Sterbebett eines Palliativpatienten ermöglicht werden (BPW)	4 (1,4)	0,10	0,70
4. Wenn man im Sterbeprozess auf eine Flüssigkeitsgabe verzichtet, spricht man von *aktiver* Sterbehilfe^a^ (PKT)	15 (5,2)	0,18	0,70
5. Morphin ist wegen der drohenden Atemdepression ungeeignet zur Therapie von Luftnot^a^	19 (6,5)^b^	0,39	0,67
6. Der in einer Patientenverfügung vorverfügte Patientenwille bezüglich einer medizinischen Maßnahme ist auch für *nicht ärztliches* Personal bindend	8 (2,7)	0,37	0,67
7. Patientenverfügungen sind aus rechtlichen Gründen nur für Hausärzte bindend	4 (1,4)	0,13	0,70
**Sie kommen als Rettungsdienst *ohne Notarzt* zu einem Einsatz, bei dem Sie einen bewusstlosen Palliativpatienten vorfinden. In seiner Patientenverfügung/seinem Palliativausweis *„lehnt der Patient ohne Einschränkung jegliche lebensverlängernden Maßnahmen ab“*. Eine Person mit Vorsorgevollmacht bestätigt Ihnen am Einsatzort, dass der Inhalt der Patientenverfügung dem aktuellen Patientenwillen entspricht.**			
8. Eine Reanimation ist im Falle eines Herz-Kreislauf-Stillstandes bis zum Eintreffen des Notarztes durchzuführen^a^	5 (1,7)	0,65	0,62
9. Eine kreislaufunterstützende medikamentöse Therapie ist erlaubt^a^	8 (2,7)	0,51	0,65
10. Eine Intubation sollte bei Atemstillstand trotzdem erfolgen^a^	7 (2,4)	0,62	0,63
11. Eine O_2_-Gabe über die Maske ist zulässig^a^	7 (2,4)	0,16	0,70
12. Eine Schmerztherapie ist erlaubt	7 (2,4)	0,22	0,69
13. Es sollte die zeitnahe Benachrichtigung einer eingebundenen SAPV (spezialisierte ambulante Palliativversorgung) erfolgen	9 (3,1)	0,33	0,68
14. Es sollte bei Bedarf ein Notfallseelsorger hinzugezogen werden	4 (1,4)	0,13	0,70
15. Bei Mitnahme sollte zuerst eine Kontaktaufnahme mit einem Krankenhaus mit Palliativstation erfolgen	4 (1,4)	0,12	0,70
**Palliative Selbstwirksamkeitserwartung: Ich bin fähig …**			
16. objektive Daten zu erheben, die das Schmerzniveau des Patienten beschreiben (BPW)	2 (0,7)	0,53	0,80
17. einem Patienten die regionalen Angebote der spezialisierten Palliativversorgung zu nennen (PKT)	1 (0,3)	0,55	0,80
18. psychosoziale Probleme zu erkennen und diese mit dem Patienten und den Angehörigen zu besprechen (PKT)	1 (0,3)	0,67	0,77
19. mit einem ängstlichen Patienten und seinen Angehörigen zu sprechen, sodass sie sich sicherer fühlen (BPW)	3 (1,0)	0,68	0,77
20. auch „schwierigen“ Patienten und Angehörigen mit Empathie zu begegnen (PKT)	1 (0,3)	0,55	0,80
21. zu erkennen, ob ein Mensch leidet, auch wenn die Möglichkeiten der Kommunikation eingeschränkt sind (PKT)	3 (1,0)	0,57	0,79

In Klammern ist die Herkunft der übernommenen Items angegeben

*RDM* Rettungsdienstmitarbeiter

^a^Umgepoltes Item

^b^Häufig nicht beantwortet

Der Kolmogorov-Smirnov-Test zeigte für keines der Items aus Tab. 1 eine Normalverteilung (*p* < 0,001).

Sowohl das Kaiser-Meyer-Olkin-Kriterium (KMO = 0,73) als auch der Bartlett-Test (Chi-Quadrat = 1311,126, *p* < 0,001) weisen darauf hin, dass sich die Items des Messinstruments für eine Faktorenanalyse eignen und Sphärizität vorliegt. Die Ergebnisse der Parallelanalyse sprechen für eine 6‑Faktoren-Lösung. Hierdurch ließen sich 50 % der Varianz erklären. Welchem Faktor die Items zugeordnet wurden, zeigt Tab. 2. Wie in Tab. 3 zu sehen ist, korrelieren, mit Ausnahme von Faktor 5, alle durch die Dimensionsreduktion gebildeten Skalen signifikant miteinander. Für Faktor 5 ließ sich eine signifikante Korrelation mit Faktor 3 errechnen.

**Table 2:** 

Item	Faktor 1	Faktor 2	Faktor 3	Faktor 4	Faktor 5	Faktor 6
19. Fähig, mit ängstlichen Patienten und Angehörigen zu sprechen, dass sie sich sicherer fühlen (BPW)	**0,8**	–	–	–	–	–
18. Fähig, psychosoziale Probleme zu erkennen und zu besprechen (PKT)	**0,7**	–	–	–	–	–
21. Fähig zu erkennen, ob ein Patient leidet, auch wenn die Möglichkeiten der Kommunikation eingeschränkt sind (PKT)	**0,7**	–	–	–	–	–
20. Fähig, auch „schwierigen“ Patienten emphatisch zu begegnen (PKT)	**0,6**	–	–	–	–	–
17. Fähig zur Nennung regionaler SAPV-Angebote (PKT)	**0,6**	–	–	–	–	–
16. Fähig zur Einschätzung des Schmerzniveaus (BPW)	**0,6**	–	–	–	–	–
10. (Fallbeispiel) Eine Intubation sollte bei Atemstillstand trotzdem erfolgen^a^	–	**0,8**	–	–	–	–
9. (Fallbeispiel) Eine kreislaufunterstützende medikamentöse Therapie ist erlaubt^a^	–	**0,8**	–	–	–	–
8. (Fallbeispiel) Eine Reanimation ist durchzuführen^a^	–	**0,7**	–	–	–	–
11. (Fallbeispiel) O_2_-Gabe über die Maske ist zulässig^a^	–	**0,4**	–	–	–	–
13. (Fallbeispiel) Es sollte die zeitnahe Benachrichtigung einer eingebundenen SAPV erfolgen	–	–	**0,7**	–	–	–
15. (Fallbeispiel) Bei Mitnahme sollte zuerst eine Kontaktaufnahme mit einem Krankenhaus mit Palliativstation erfolgen	–	–	**0,5**	–	–	–
12. (Fallbeispiel) Eine Schmerztherapie ist erlaubt	–	–	**0,4**	–	–	–
14. (Fallbeispiel) Es sollte bei Bedarf ein Notfallseelsorger hinzugezogen werden	–	–	**0,4**	–	–	–
2. Bei vorbestehender Therapie mit Opioiden ist die zusätzliche Gabe von Sedativa wegen Gefahr der Atemdepression kontraindiziert^a^	–	–	–	**0,6**	–	–
5. Morphin ist wegen der drohenden Atemdepression ungeeignet zur Therapie von Luftnot^a^	–	–	–	**0,3**	–	–
4. Wenn man im Sterbeprozess auf eine Flüssigkeitsgabe verzichtet, spricht man von *aktiver* Sterbehilfe^a^ (PKT)	–	–	–	**0,3**	–	–
7. Patientenverfügungen sind aus rechtlichen Gründen nur für Hausärzte bindend^a^	–	–	–	–	**0,5**	**–**
3. Familienmitgliedern sollte die Anwesenheit am Sterbebett eines Palliativpatienten ermöglicht werden (BPW)	–	–	–	–	**0,5**	**–**
1. Palliativpatienten sollte die Wahrheit nicht vorenthalten werden, damit sie sich auf den Tod vorbereiten können (BPW)	–	–	–	–	**0,4**	**–**
6. Der in der Patientenverfügung vorverfügte Wille ist auch für nicht ärztliches Personal bindend^a^	–	**–**	–	–	–	**0,4**

In Klammern ist die Herkunft der übernommenen Items angegeben

^a^Umgepoltes Item

**Table 3:** 

		Faktor 1	Faktor 2	Faktor 3	Faktor 4	Faktor 5	Faktor 6
Faktor 1	Korrelation	1	0,30**	0,13*	0,13*	0,03	0,21**
	Signifikanz	–	0,001	0,019	0,026	0,625	0,001
Faktor 2	Korrelation	0,30**	1	0,13*	0,28**	−0,01	0,42**
	Signifikanz	0,001	–	0,027	0,001	0,835	0,001
Faktor 3	Korrelation	0,13*	0,13*	1	0,21**	0,12*	0,26**
	Signifikanz	0,019	0,027	–	0,001	0,043	0,001
Faktor 4	Korrelation	0,13*	0,29**	0,21**	1	0,04	0,13*
	Signifikanz	0,026	0,001	0,001	–	0,530	0,023
Faktor 5	Korrelation	0,03	−0,01	0,12*	0,04	1	0,07
	Signifikanz	0,625	0,835	0,043	0,530	–	0,258
Faktor 6	Korrelation	0,21**	0,42**	0,26**	0,13*	0,07	1
	Signifikanz	0,001	0,001	0,001	0,023	0,258	–

*Die Korrelation ist auf einem Niveau von 0,05 (2-seitig) signifikant

**Die Korrelation ist auf einem Niveau von 0,01 (2-seitig) signifikant

**Figure 1:**
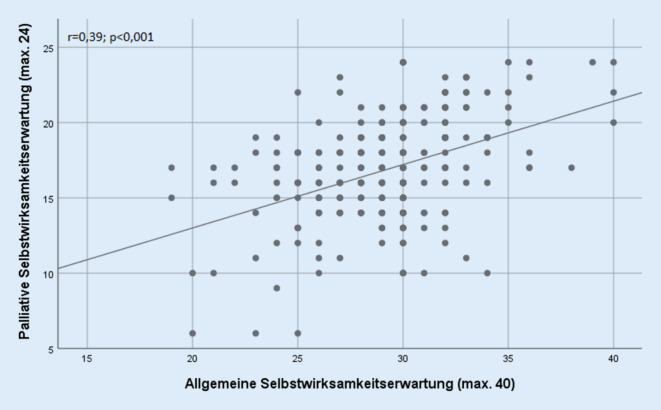

Es ergibt sich eine signifikante statistische Korrelation zwischen der etablierten allgemeinen Selbstwirksamkeitserwartungsskala nach Schwarzer und Jerusalem und der palliativen Selbstwirksamkeitserwartungsskala. Der Spearman-Korrelationskoeffizient beider Skalen liegt bei r = 0,39, *p* < 0,001. Die Korrelation der beiden Skalen (allgemeine Selbstwirksamkeitserwartung und palliative Selbstwirksamkeitserwartung) ist grafisch in Abb. 1 dargestellt.

Bei der Messung der Kriteriumsvalidität zeigte sich ein signifikanter Unterschied im palliativen Wissen zwischen den Palliative-Care-Pflegekräften (M_Rang_ 289,73) und den Rettungsdienstmitarbeitern (M_Rang_ 146,97, U = 281.000, r = 0,40, *p* < 0,001).

Die interne Konsistenz durch Berechnung von Cronbachs α ergab für alle Wissensfragen ein α = 0,70 und für die Subskala der palliativen Selbstwirksamkeitserwartung ein α = 0,82. Der Beitrag der einzelnen Items zur Gesamtkonsistenz des Messinstruments sowie die Trennschärfe sind in Tab. 1 dargestellt.

Vier Items (Tab. 4) wurden nicht selektiert und aus dem endgültigen Fragebogen entfernt.

**Table 4:** 

	Von den RDM nicht beantwortet, *n* (%)	Trennschärfe	Reliabilität „α if item deleted“
Ein Palliativpatient ist ein Patient mit nur noch begrenzter Lebenserwartung	2 (0,7)	0,07	0,69
Opioide können mit Nicht-Opioiden(z. B. Novalgin®, Paracetamol) zur Schmerztherapie kombiniert werden	22 (7,6)^b^	0,20	0,69
Das Anheben der Raumtemperatur lindert häufig die Atemnot^a^ (PKT)	10 (3,4)	0,17	0,69
Für eine Therapieentscheidung braucht es neben einer medizinischen Indikation auch immer die mutmaßliche Zustimmung des Patienten	4 (1,4)	0,05	0,70

In Klammern ist die Herkunft der übernommenen Items angegeben

*RDM* Rettungsdienstmitarbeiter

^a^Umgepoltes Item

^b^Häufig nicht beantwortet

## Diskussion

Bereits in früheren Veröffentlichungen wurde auf die Bedeutung palliativmedizinischer Schulungen für eine kompetente Versorgung von Palliativpatienten durch die Rettungsdienste hingewiesen [22, 25, 27]. Denn an der Betreuung von Palliativpatienten sind neben Ärzten und Pflegekräften nicht zuletzt auch die Mitarbeiter der Rettungsdienste beteiligt [21, 24]. Diese Studie stellt die Entwicklung und Validierung des ersten deutschsprachigen Testinstruments für palliatives Wissen und Selbstwirksamkeitserwartung im Rettungsdienst vor.

Mithilfe des Delphi-Prozesses wurden in mehreren Runden die Inhalte und Bestandteile des Testinstruments definiert, was insgesamt für eine gute Inhaltsvalidität spricht [11]. Vonseiten des Rettungsdienstes wurde die Meinung vertreten, dass Items zu den Themen medikamentöse Analgesie und Dyspnoebehandlung ausschließlich in den ärztlichen Kompetenzbereich fallen und daher für die Rettungsdienstmitarbeiter von untergeordneter Relevanz seien. Hier kamen die Experten während des Delphi-Prozesses allerdings zu dem Konsens, dass auch diese Themengebiete besonders im Zuge der Einführung der Notfallsanitäterausbildung und der damit einhergehenden erweiterten Kompetenzen für den Rettungsdienst an Bedeutung gewinnen [3, 12]. Dieser Umstand spiegelt sich auch darin wider, dass Items zu den Themen Analgesie und Dyspnoebehandlung am häufigsten nicht beantwortet wurden (Tab. 1). Aber schon eine Expertengruppe, die sich auf Einladung der Arbeitsgemeinschaft Südwestdeutscher Notärzte (agswn) und der Klinik für Anästhesiologie der Universitätsmedizin der Johannes Gutenberg-Universität in Mainz zusammenfand, stellte klar, dass nichtärztliches Rettungsdienstfachpersonal bei einer Medikamentengabe zum Beispiel im Rahmen von standardisierten Vorgaben (SOP) sicher in der Indikationsstellung und dem Umgang mit Nebenwirkungen sein muss [7, 15]. Hier sollten weiterführende Gespräche und Untersuchungen folgen.

Die Vielschichtigkeit von palliativem Wissen zeigt sich in der Konstruktvalidierung (Faktorenanalyse). Während die Items der „palliativen Selbstwirksamkeitserwartung“ (Faktor 1) durch die Analyse einem gemeinsamen Faktor zugeordnet werden, lassen sich die Items, die palliatives Wissen abfragen sollen, nochmals auf 5 Faktoren bzw. Skalen differenzieren (Tab. 2). Diese Skalen lassen sich gliedern in: „Handeln und Entscheidung in der Palliativversorgung“ (Faktor 2), „Kenntnis der Strukturen in der Palliativversorgung“ (Faktor 3), „medikamentöse und nichtmedikamentöse Symptombehandlung“ (Faktor 4), „end-of-life care“ (Faktor 5) und „rechtliche Thematik“ (Faktor 6). Sie spiegeln damit auch die Themengebiete wider, die zuvor von den Experten im Delphi-Prozess als Inhalte des Fragebogens festgelegt wurden. Es ist anzumerken, dass die Items 7 und 12, welche sich mit rechtlicher Thematik und Schmerztherapie befassen, thematisch nicht zum Faktor 5 bzw. 3 passen. Trotzdem wurde hier auf die Entfernung dieser Items verzichtet, um die inhaltliche Breite des Messinstruments und Inhaltsvalidität nicht zu gefährden. Alle gebildeten Skalen stellen die Anforderungen an die Arbeit im palliativen Kontext dar, was auch die signifikanten Korrelationen der verschiedenen Faktoren miteinander zeigen. Dass es hierbei aber Einschränkungen gibt und Faktor 5 ausschließlich mit Faktor 3 („Kenntnis der Strukturen in der Palliativversorgung“) signifikant korreliert, kann damit erklärt werden, dass es sich bei den Skalen nicht um natürliche Dimensionen wie z. B. Persönlichkeitseigenschaften oder einen Symptomcluster, sondern um eine praktisch-pragmatisch zusammengesetzte Wissensdomäne handelt.

Die statistische Korrelation von allgemeiner und palliativer Selbstwirksamkeitserwartung liefert im Sinne der konvergenten Validität weiterhin einen wichtigen Beitrag zur Güte des Messinstruments. Die moderate Korrelation [4] könnte dahingehend interpretiert werden, dass allgemeine und palliative Selbstwirksamkeitserwartung nur bedingt miteinander vergleichbar sind. Zusätzlich wurden die Scores der Rettungsdienstmitarbeiter im palliativen Wissen mit denen von Palliative-Care-Pflegekräften verglichen. Der signifikante Gruppenunterschied mit einer Effektstärke von r = 0,40 spricht für eine bestehende Kriteriumsvalidität [8].

Die Messung der Reliabilität mittels Cronbachs α als Maß der internen Konsistenz ergab für die Subskala „palliatives Wissen“ einen akzeptablen Wert. Die Reliabilität kann auch durch Entfernung weiterer Items nicht erhöht werden (Tab. 1). Um die Akzeptanz (und somit Adhärenz) des Tests bei den Rettungsdienstmitarbeitern hoch zu halten, wurde versucht, den Fragebogen mit 19 Items möglichst kurz zu halten und alle vorher festgelegten Themengebiete abzudecken. Doch die daraus resultierende Zunahme der inhaltlichen Heterogenität der Skala führt zur Beeinträchtigung der internen Konsistenz, wie schon Rammstedt berichtet [20]. Die interne Konsistenz der Subskala „palliative Selbstwirksamkeitserwartung“ ist als gut zu bewerten [1].

Für mehrere Items des Wissenstests war die Trennschärfe < 0,3. Diese Items wurden gleichfalls aus Gründen der inhaltlichen Breite belassen, auch um möglichst alle von den Experten vorgegebenen Themenbereiche im Test zu repräsentieren. Außerdem könnte die teils niedrige Korrelation dieser Items mit dem Gesamttestwert ein weiteres Indiz für die Vielschichtigkeit von „palliativem Wissen“ sein und damit erklärt werden, dass sehr unterschiedliche Teilbereiche palliativen Wissens mit dem vorliegenden Test untersucht werden. Beispielhaft wären hier die Items 1 und 6 (Tab. 1) zu nennen, die mit Spiritualität und rechtlichen Aspekten doch sehr unterschiedliche Themengebiete abdecken. Schon den Autoren des Bonner Palliativwissenstests und des palliativen Kompetenztests für Ärzte fielen bei der Validierung ihrer Skalen zum palliativen Wissen teils niedrigere Trennschärfen der Items auf, sie erklärten diese mit der Komplexität von „palliativem Wissen“ [16, 18]. Um dies abschließend klären zu können, bedarf es allerdings weiterer Untersuchungen. Alle Items der Subskala „palliative Selbstwirksamkeitserwartung“ entsprachen den Anforderungen an eine gute Trennschärfe [6], was insgesamt auf einen homogeneren Testteil schließen lässt. Diese Subskala beabsichtigt, die Umsetzung des „palliativen Wissens“ bzw. das Verhalten in der alltäglichen Versorgung von Palliativpatienten widerzuspiegeln und zu messen, ob die Rettungsdienstmitarbeiter auch in schwierigen Situationen in der Lage sind, gelerntes Wissen umzusetzen [9]. Denn während Wissensfragen eher reines Faktenwissen abfragen, zielen die Items zur Selbstwirksamkeitserwartung darauf ab, fachliches Handeln abzubilden [23].

Vier Items wurden nicht selektiert und aus dem Fragebogen entfernt (Tab. 4), weil sie in der Faktorenanalyse keinem Faktor zugeordnet werden konnten.

Aus- und Weiterbildungen sind essenziell für den Kompetenzgewinn. Während der Begriff „Kompetenz“ im Rettungsassistentengesetz (RettAssG; [14]) noch keine Rolle spielte, ist er von zentraler Bedeutung für das dem Rettungsassistentengesetz folgende Notfallsanitätergesetz (NotSanG; [17, 19]). Beispielsweise sollen Auszubildende im Rettungsdienst die Kompetenz erwerben, verschiedenste Lebens- und Gefühlslagen sowie Probleme von Patienten und Angehörigen wahrzunehmen, diese zu bewerten und ihr Handeln anzupassen. Dies deckt sich auch mit den Kernkompetenzen 3 und 8, die von der Arbeitsgruppe Bildung der DGP im KoMPaC für die palliative Lehre vorgeschlagen werden [30]: Sie befassen sich mit den psychosozialen Bedürfnissen der Patienten und den Anforderungen an die Versorgungskoordination, die bei allen Settings an die Palliativversorgung gestellt werden. Zusammenfassend wird hierdurch die Bedeutung eines Tests zur Überprüfung der Bildungsmaßnahmen zusätzlich hervorgehoben. Denn mit diesem validierten Test liegt derzeitig das einzige Instrument vor, mit dem Fortschritte durch Aus- und Weiterbildung nachgewiesen werden können. Somit könnte er auch als Diskussionsgrundlage für die Entwicklung eines entsprechenden Tests in der notärztlichen Tätigkeit dienen.

## Limitationen

Da die Teilnahme an der Untersuchung freiwillig im Rahmen von allgemeinen rettungsdienstlichen Fortbildungen war, ist davon auszugehen, dass überwiegend motivierte Rettungsdienstmitarbeiter teilnahmen, die zusätzlich ein besonderes Interesse an der Palliativmedizin haben. Dies spiegelt sich sicherlich auch in der Rekrutierungsquote von 39 % als Selektionseffekt wider. Ungeachtet dessen konnte ein signifikanter Effekt zwischen Rettungsdienstmitarbeitern und Palliative-Care-Pflegekräften gefunden werden. Obwohl der Testbogen auch auf Wunsch des Rettungsdienstes möglichst kurz gefasst wurde, umfasste er trotzdem noch 64 Items. Auch diese Tatsache könnte zu einer geringeren Teilnahmemotivation beigetragen haben. Daher scheint die Stichprobe nicht vollumfänglich mit der Grundgesamtheit vergleichbar zu sein. Es handelt sich bei der vorliegenden Studie um die erste Konstruktion und Validierung eines palliativen Wissenstests mit Messung der palliativen Selbstwirksamkeitserwartung im Rettungsdienst. Weitere Validierungen dieses Tests sind auch aufgrund der o. g. Limitationen wünschenswert.

## Fazit für die Praxis

Mit der Entwicklung und Validierung von PARPACT liegt nun ein Messinstrument vor, mit dem Fortschritte von Aus- und Weiterbildungsmaßnahmen im Rettungsdienst überprüft werden können. Vor dem Hintergrund der Notfallsanitäterausbildung, durch die die Rettungsdienstmitarbeiter zusätzliche Kompetenzen erhalten, nimmt der Stellenwert von Weiterbildungen nochmals zu. Gleichzeitig ist es umso wichtiger, die Lernerfolge zu evaluieren. Hierfür sowie auch für die Überprüfung des Effekts der Palliativ- bzw. Notfallausweise kann der vorliegende Test genutzt werden. Außerdem ist ein Ausbau des Tests durch Ergänzung weiterer Items denkbar.
